# *Bacillus subtilis* produces (p)ppGpp in response to the bacteriostatic antibiotic chloramphenicol to prevent its potential bactericidal effect

**DOI:** 10.1002/mlf2.12031

**Published:** 2022-06-30

**Authors:** Jin Yang, Jessica T. Barra, Danny K. Fung, Jue D. Wang

**Affiliations:** ^1^ Department of Bacteriology University of Wisconsin Madison USA

**Keywords:** antibiotic tolerance, bactericidal antibiotic, bacteriostatic antibiotic, GTP, (p)ppGpp

## Abstract

Antibiotics combat bacteria through their bacteriostatic (by growth inhibition) or bactericidal (by killing bacteria) action. Mechanistically, it has been proposed that bactericidal antibiotics trigger cellular damage, while bacteriostatic antibiotics suppress cellular metabolism. Here, we demonstrate how the difference between bacteriostatic and bactericidal activities of the antibiotic chloramphenicol can be attributed to an antibiotic‐induced bacterial protective response: the stringent response. Chloramphenicol targets the ribosome to inhibit the growth of the Gram‐positive bacterium *Bacillus subtilis*. Intriguingly, we found that chloramphenicol becomes bactericidal in *B. subtilis* mutants unable to produce (p)ppGpp. We observed a similar (p)ppGpp‐dependent bactericidal effect of chloramphenicol in the Gram‐positive pathogen *Enterococcus faecalis*. In *B. subtilis*, chloramphenicol treatment induces (p)ppGpp accumulation through the action of the (p)ppGpp synthetase RelA. (p)ppGpp subsequently depletes the intracellular concentration of GTP and antagonizes GTP action. This GTP regulation is critical for preventing chloramphenicol from killing *B. subtilis*, as bypassing (p)ppGpp‐dependent GTP regulation potentiates chloramphenicol killing, while reducing GTP synthesis increases survival. Finally, chloramphenicol treatment protects cells from the classical bactericidal antibiotic vancomycin, reminiscent of the clinical phenomenon of antibiotic antagonism. Taken together, our findings suggest a role of (p)ppGpp in the control of the bacteriostatic and bactericidal activity of antibiotics in Gram‐positive bacteria, which can be exploited to potentiate the efficacy of existing antibiotics.

## Impact statement

The antimicrobial action of antibiotics can be broadly classified as bacteriostatic (i.e., inhibits growth) or bactericidal (i.e., lethality), which is largely considered as a property of the antibiotic. Here, we report that the Gram‐positive model bacterium *Bacillus subtilis* produces the signaling nucleotide (p)ppGpp in response to the bacteriostatic ribosome‐targeting antibiotic chloramphenicol. In the absence of this response, chloramphenicol mediates a strong bactericidal action. The description of the (p)ppGpp‐dependent bacteriostatic–bactericidal switch and its molecular mechanisms documented in this study provides a new insight into the understanding of antimicrobial properties, which can inspire the future development of antibacterial therapy by targeting bacterial nucleotide signaling.

## INTRODUCTION

The discovery of antimicrobial compounds is a historical advancement in the treatment of infectious diseases^1^. Despite their diverse chemical structures and mechanisms of action, different antibiotics can be broadly classified into two categories based on their effect on the microbes: bactericidal or bacteriostatic antibiotics^2^. Under laboratory conditions, bactericidal antibiotics have a minimum inhibitory concentration (MIC) that is close to their minimum bactericidal concentration^3^, demonstrating their lethal effects to bacteria. In contrast, bacteriostatic antibiotics inhibit bacterial growth at MIC and only exert bactericidal effects at much higher concentrations, if at all^3^, indicating that their primary action is to halt bacterial growth.

The differences in the pharmacodynamic properties between bacteriostatic and bactericidal antibiotics can be attributed to multiple factors^4^. For example, it has been proposed that immediate actions of bactericidal antibiotics such as beta‐lactams, quinolones, and aminoglycosides involve corruption of essential cellular processes such as cell wall synthesis, DNA replication, and translation, thus causing irreversible damage and cell death^5^. On the other hand, bacteriostatic antibiotics such as macrolides, tetracyclines, and chloramphenicol appear to inhibit cellular metabolism to stop bacterial cell growth (stasis), thus preserving the viability and ability to regrow after the drug is removed^6^, ^7^. However, it is known that certain antibiotics that are bacteriostatic can be bactericidal when applied to different bacterial species^2^. For example, the 50S ribosome‐targeting antibiotic chloramphenicol, which is a well‐known bacteriostatic antibiotic that arrests growth in most species of bacteria, is not uniformly bacteriostatic and can be bactericidal for related bacterial species^8^. Therefore, it remains incompletely understood whether the drug effects on growth versus viability are due to basic mechanistic differences between the drugs and whether the differences between bacteriostatic and bactericidal effects can be attributed to intrinsic or responsive protective mechanisms among bacterial species^4^.

Bacteria show extensive responses to bactericidal antibiotics from subinhibitory to inhibitory concentrations^9^. DNA‐damaging antibiotics such as ciprofloxacin can induce the SOS response, which promotes DNA repair^10^, ^11^. Cell wall antibiotics such as vancomycin (VAN) can induce the cell envelope stress response, activating regulons of alternative sigma factors^12^, ^13^. These responses can either decrease or increase the efficacy of the antibiotics. For example, bacterial response to bacitracin can induce efflux of the antibiotics, thus increasing antibiotic resistance^14^. The SOS response can increase persistence or tolerance to DNA‐damaging antibiotics^15^, ^16^. On the other hand, bacterial responses such as altered redox response and reactive oxygen species (ROS) generation have been reported to contribute to or be considered to be the major causes of killing by bactericidal antibiotics^17^.

In contrast to these specific responses to treatment by bactericidal antibiotics, there have been few reports of bacterial responses triggered by bacteriostatic antibiotics, as it is believed that cells show only mild responses other than the arrest of macromolecular synthesis. In terms of the bacteriostatic antibiotic chloramphenicol, transcription response and proteomic alteration after treatment by chloramphenicol have been characterized in the Gram‐positive bacterium *Bacillus subtilis* ^18^, ^19^. However, their relevance to the efficacy of antibiotic treatment is unexplored.

In this study, we characterized an unexpected stress response triggered by chloramphenicol treatment in *B. subtilis* and revealed its key function in protecting bacterial survival against the antibiotic. The stringent response is a nutritional stress response in bacteria that is mediated by the accumulation of the nucleotide alarmones pppGpp and ppGpp, together called (p)ppGpp. Strikingly, in the absence of (p)ppGpp synthesis by the enzyme RelA, the bacteriostatic antibiotic chloramphenicol becomes strongly bactericidal in *B. subtilis*. We then delineated this pathway by epistatic analyses to identify that (p)ppGpp protects cells from chloramphenicol‐induced death by controlling GTP, which is a conserved role of (p)ppGpp in Gram‐positive bacteria. Our findings suggest that *B. subtilis* harnessed (p)ppGpp signaling to render an otherwise bactericidal antibiotic response bacteriostatic. This chloramphenicol protection by (p)ppGpp is not observed in the Gram‐negative bacterium *Escherichia coli*, but is observed in the opportunistic pathogen *Enterococcus faecalis*, implying that different stress responses to antibiotics in different species are important determinants of antibiotic treatment outcomes.

## RESULTS

### Chloramphenicol is bactericidal in Gram‐positive bacterial mutants defective in the stringent response

**Figure 1:**
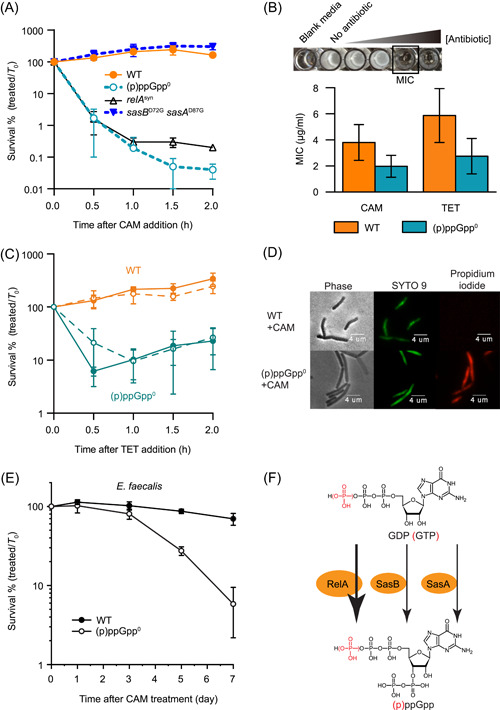
The bacteriostatic antibiotic chloramphenicol (CAM) is bactericidal to cells lacking (p)ppGpp synthesis. (A) Exponentially growing *B. subtilis* SMY wild type (WT), (p)ppGpp^0^, *relA* ^syn^, and *sasB* ^D72G^ *sasA* ^D87G^ were treated with 12 μg/ml CAM (4× minimum inhibitory concentration [MIC]) for up to 2 h. (B) MIC measurement of CAM and tetracycline (TET). Values are means (*N* ≥ 11) ± SD. (C) *Bacillus subtilis* SMY WT and (p)ppGpp^0^ exponentially growing in LB (solid line) or S7 medium (dashed line) were treated with 0.5 μg/ml tetracycline (TET) (0.06× MIC) for up to 2 h. (A, C) Percent survival was determined by dividing the number of CFU/ml at each time point by the number of CFU/ml at *T* = 0 and converted into a percentage. Values are means (*N* = 3) ± SD. (D) Wild‐type and (p)ppGpp^0^ cells were treated with 12 μg/ml CAM for 5 h and stained with SYTO 9 and propidium iodide. Phase‐contrast, SYTO 9 fluorescence, and propidium iodide fluorescence microscopic pictures are shown. (E) Percent survival of *Enterococcus faecalis* OG1RF wild type and (p)ppGpp^0^ mutant after 25 μg/ml CAM treatment. Values are means (*N* = 3) ± SD. (F) Schematics of three (p)ppGpp synthetases in *B. subtilis*. RelA is the major (p)ppGpp synthetase in response to amino acid starvation. SasB synthesizes basal‐level (p)ppGpp. SasA is induced by cell wall stress and synthesizes (p)ppGpp.

Chloramphenicol is a broad‐spectrum antibiotic that targets the 50S ribosome to inhibit peptidyl transfer^20^, ^21^. Chloramphenicol is generally considered a prime example of a bacteriostatic antibiotic in most bacteria, including the Gram‐negative bacterium *E. coli* and the Gram‐positive bacterium *B. subtilis*. The stringent response is mediated by intracellular accumulation of (p)ppGpp and promotes bacterial survival during nutrient stress. We found, unexpectedly, that (p)ppGpp is necessary for the survival of *B. subtilis* cells during chloramphenicol treatment. Chloramphenicol treatment at ~4× MIC rapidly inhibited the growth of wild‐type cells, with no detectable loss in viability (Figure 1A), as was reported before^22^, ^23^. In contrast, when we applied chloramphenicol to a (p)ppGpp^0^ mutant devoid of all three of its (p)ppGpp synthetase genes, *relA*, *sasB*, and *sasA*, chloramphenicol was highly bactericidal and killed the vast majority of (p)ppGpp^0^ cells rapidly, with an ~99.9% reduction in viable counts within 2 h of treatment (Figure 1A). This strong bactericidal effect was not due to a change in MIC, as (p)ppGpp^0^ cells showed only a modest change in MIC (Figure 1B). This effect is not limited to chloramphenicol, as the 30S ribosome inhibitor tetracycline also become bactericidal to (p)ppGpp^0^ cells (Figure 1C).

To examine whether the reduction in viable counts is due to loss of cultivability through the formation of viable but nonculturable‐state bacteria^24^, ^25^, or reflects cell death, we visualized chloramphenicol‐treated cells with microscopy using live death staining (Figure 1D). We found that after chloramphenicol treatment, wild‐type cells were only stained with SYTO 9, a low‐affinity nucleic acid dye that can enter live cells. In contrast, a large fraction of (p)ppGpp^0^ cells was stained by high‐affinity nucleic acid stain propidium iodide, which can penetrate only a permeabilized cell envelope, confirming that chloramphenicol treatment resulted in cell death in the absence of (p)ppGpp.

Although (p)ppGpp is a conserved nucleotide in bacteria, its role in chloramphenicol protection is species‐dependent. Chloramphenicol‐mediated killing is not observed in the well‐characterized Gram‐negative bacterium *E. coli*, as mutants defective in (p)ppGpp production remain viable upon chloramphenicol treatment^26^. We sought to investigate whether the role of (p)ppGpp on chloramphenicol survival is restricted to *B. subtilis* or applies to related Gram‐positive bacteria such as the opportunistic pathogen *E. faecalis*. We first measured the MIC of chloramphenicol in *E. faecalis*. In the brain–heart infusion (BHI) growth medium, both the wild‐type and the (p)ppGpp^0^ mutant of *E. faecalis* have a MIC of 8 μg/ml, which is similar to the previous report (4 μg/ml)^27^. Next, we measured the survival of wild‐type *E. faecalis* and its (p)ppGpp^0^ mutant after ~3× MIC chloramphenicol treatment (Figure 1E). While chloramphenicol remained bacteriostatic in wild‐type *E. faecalis* even after prolonged treatment, it exerted a strong bactericidal effect in the (p)ppGpp^0^ mutant after 3 days of treatment (Figure 1E). The slower killing of the *E. faecalis* (p)ppGpp^0^ mutant compared to *B. subtilis* suggests that *E. faecalis* may have additional protective mechanisms that delayed chloramphenicol lethality. Nevertheless, this finding suggests that (p)ppGpp is essential for surviving chloramphenicol treatment beyond *B. subtilis*.

### *Bacillus subtilis* produces (p)ppGpp in response to chloramphenicol treatment through RelA

**Figure 2:**
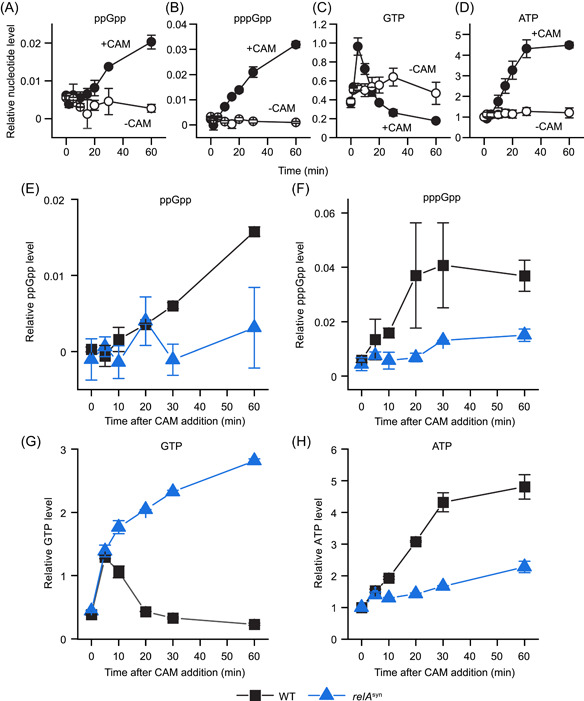
*Bacillus subtilis* produces (p)ppGpp in response to chloramphenicol (CAM) treatment through RelA. (A–D) ^32^P‐orthophosphate radiolabeled *Bacillus subtilis* SMY wild‐type cells were treated with (closed circles) and without (open circles) 12 μg/ml CAM and nucleotides were extracted at *T* = 0 to 60 min. ppGpp (A), pppGpp (B), GTP (C), and ATP (D) levels were determined and normalized to the ATP level at *T* = 0 and OD_600_ relative to *T* = 0. Values are means (*N* = 2) ± SEM. (E–H) Thin‐layer chromatography (TLC) measurements of ppGpp (E), pppGpp (F), GTP (G), and ATP (H) levels normalized to relative OD_600_ to time zero and divided by the ATP level at *T* = 0 in *B. subtilis* NCIB3610 wild‐type (WT) and *relA* ^syn^ cells treated with 12 μg/ml CAM. Values are means (*N* = 2) ± SEM.

*Bacillus subtilis* contains three (p)ppGpp synthetases (Figure 1F): a ribosome‐associated bifunctional enzyme RelA that can both synthesize and hydrolyze (p)ppGpp^28^, and two small alarmone synthetases SasB (also called YjbM/RelQ/SAS1) and SasA (also called YwaC/RelP/SAS2)^29^, ^30^. To determine which of the three (p)ppGpp synthetases in *B. subtilis* is necessary for chloramphenicol survival, we generated mutants with inactivated synthetase activities^28^, ^31^, and then assessed their survival after chloramphenicol treatment. We found that inactivation of (p)ppGpp synthesis by RelA (*relA* ^syn^) resulted in cell death resembling that of (p)ppGpp^0^, indicating that RelA synthetase activity is necessary to survive chloramphenicol treatment (Figure 1A). On the other hand, inactivation of SasB (*sasB* ^D72G^) and SasA (*sasA* ^D87G^) only resulted in bacteriostasis similarly to wild‐type cells. These results together demonstrate that the (p)ppGpp synthetase RelA is necessary and sufficient for chloramphenicol survival.

(p)ppGpp exists within *B. subtilis* cells at the basal level (~10 µM) in homeostatic growth or accumulates to ~1–2 mM upon amino acid starvation^32^. We found that chloramphenicol can also induce (p)ppGpp in *B. subtilis* (Figure 2), in agreement with an early report in 1975^33^. Chloramphenicol treatment induced accumulation of both ppGpp and pppGpp in wild‐type cells (Figure 2A,B) to an estimated level of ~100 μM, which was ~10‐fold higher than that in untreated cells, although ~10‐fold lower than 1–2 mM levels of (p)ppGpp during amino acid starvation (Figure 2A,B). Importantly, neither ppGpp nor pppGpp was induced by chloramphenicol in the *relA* ^syn^ strain (Figure 2E,F), indicating that the nucleotides produced in response to chloramphenicol are indeed products of the RelA (p)ppGpp synthetase. Together, our data suggest that chloramphenicol‐induced (p)ppGpp synthesis by RelA promotes survival to chloramphenicol treatment.

### (p)ppGpp downregulates GTP levels to prevent chloramphenicol lethality

How does (p)ppGpp prevent chloramphenicol from killing *B. subtilis*? In *B. subtilis*, (p)ppGpp regulates many cellular processes, including DNA replication^34^, ^35^, transcription^36^, ribosome biogenesis^37^, ^38^ and protein translation and secretion^39^ ^–^ ^41^. However, its most well‐characterized effect in Gram‐positive bacteria is the regulation of purine metabolism^31^, ^36^, ^37^, ^42^ ^–^ ^47^. In *B. subtilis*, (p)ppGpp inhibits purine biosynthesis enzymes GuaB, Gmk, HprT, and XprT, with a strong impact on GTP levels and viability^32^. We observed that upon chloramphenicol treatment, GTP levels immediately increased, presumably because translation is powered by GTP, and translation inhibition by chloramphenicol strongly reduced GTP consumption (Figure 2C). Importantly, as (p)ppGpp accumulated, GTP levels subsequently decreased to a level that was even lower than that of untreated cells (Figure 2C). This GTP depletion can be explained by (p)ppGpp‐mediated inhibition of GTP synthesis^32^. Strikingly, in the *relA* ^syn^ mutant, the GTP level continued to increase to much higher levels in response to chloramphenicol treatment (Figure 2G). These results provided the possibility that high GTP during chloramphenicol treatment contributes to its killing, and regulation of GTP by (p)ppGpp contributes to the prevention of chloramphenicol lethality. Although ATP concentrations also increased upon chloramphenicol treatment in wild‐type cells (Figures 2D,H), the ATP level in *relA* ^syn^ was similar to that of untreated cells and therefore unlikely to contribute to cell death. We next used genetic analysis to examine whether there is a direct causal relationship between GTP alteration and chloramphenicol survival.

**Figure 3:**
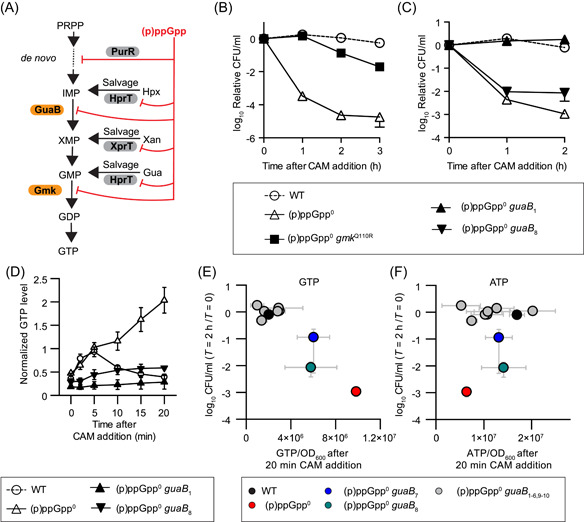
(p)ppGpp protects against chloramphenicol (CAM)‐mediated killing by reducing GTP. (A) Schematic of GTP biosynthesis regulation by (p)ppGpp in *Bacillus subtilis*. (B–C) Exponential‐phase *B. subtilis* 3610 pBS32− wild type (WT), (p)ppGpp^0^, (p)ppGpp^0^ *gmk* ^Q110R^ (B), and *B. subtilis* YB886 wild type, (p)ppGpp^0^, and (p)ppGpp^0^ *guaB* mutants (C) were treated with 12 μg/ml CAM. The relative CFU/ml was determined by dividing the number of CFU/ml at each time point by the number of CFU/ml at *T* = 0. Values are means (*N* ≥ 3) ± SEM. (D) ^32^P‐orthophosphate radiolabeled *B. subtilis* YB886 wild type, (p)ppGpp^0^, and (p)ppGpp^0^ *guaB* mutants (*guaB*_1_ and *guaB*_8_) were treated with 12 μg/ml CAM, and nucleotides were extracted at *T* = 0 to 20 min. GTP levels were determined and normalized to the ATP level and OD_600_ at *T* = 0. Values are means (*N* = 2) ± SEM. (E, F) Exponential‐phase *B. subtilis* YB886 wild type, (p)ppGpp^0^, and (p)ppGpp^0^ *guaB* mutants (*guaB*_1_ to *guaB*_10_) were treated with 12 μg/ml CAM for 2 h. Their survival was plotted against GTP levels (E) and ATP levels (F) normalized to OD_600_ after 20 min of 12 μg/ml CAM treatment obtained by ^32^P‐orthophosphate labeling in (D). Values are means (*N* ≥ 2) ± SEM.

We first examined whether GTP downregulation is sufficient to bypass the (p)ppGpp requirement for chloramphenicol survival. To reduce GTP synthesis in the absence of (p)ppGpp, we isolated a mutant with a partial loss‐of‐function mutation in the gene *gmk*, which encodes guanylate kinase responsible for the production of GDP from GMP (Figure 3A). This mutant was obtained by saturating a genetic selection for the suppressors of (p)ppGpp^0^ that rescue its amino acids auxotrophy^32^. The resulting (p)ppGpp^0^ *gmk* ^Q110R^ mutant shows a strongly reduced GTP concentration compared to the (p)ppGpp^0^ mutant^48^. We found that upon chloramphenicol treatment, the mutant showed a robust survival similar to the wild‐type cells (Figure 3B). This result suggests that lowering the GTP level is sufficient for stasis and prevention of chloramphenicol lethality. If this is true, we would expect that mutations in genes other than *gmk* that reduce GTP synthesis can also protect (p)ppGpp^0^ cells against chloramphenicol lethality. In addition to *gmk*, we previously isolated suppressors of (p)ppGpp^0^ that mapped to multiple alleles of the gene *guaB* encoding the enzyme IMPDH (Figure 3A), all of which lead to reduced GTP levels in (p)ppGpp^0^ cells^32^, ^45^, ^48^. Treatments of (p)ppGpp^0^ *guaB*_*1*_ and (p)ppGpp^0^ *guaB*_*8*_ strains with chloramphenicol resulted in higher survival compared to (p)ppGpp^0^ (Figure 3C), while the intracellular GTP levels in both suppressors during chloramphenicol treatments were much lower than those of (p)ppGpp^0^ cells (Figure 3D), confirming that lowering GTP can bypass (p)ppGpp requirement in rescuing chloramphenicol lethality.

Intriguingly, the extent of the protection varies between the two strains: chloramphenicol mediates complete stasis in (p)ppGpp^0^ *guaB*_*1*_, but significant lethality in (p)ppGpp^0^ *guaB*_*8*_. Comparison of the intracellular GTP levels of these strains during the chloramphenicol treatment (Figure 3D) revealed that (p)ppGpp^0^ *guaB*_*1*_ has the lowest GTP levels, while in (p)ppGpp^0^ *guaB*_*8*_, GTP levels are less reduced, suggesting that the degrees of protection against chloramphenicol correlate with that of GTP levels. To examine the correlation more quantitatively, we utilized the entire set of (p)ppGpp^0^ *guaB* suppressor strains with different GTP levels to examine the correlation between GTP levels and chloramphenicol lethality. They showed a range of survival that was inversely correlated to their respective GTP levels (Figure 3E). As expected, we found no correlation between chloramphenicol survival and ATP levels (Figure 3F). These results together indicated that lower GTP levels mediate stasis and prevent lethality upon chloramphenicol treatment and the degree of protection is dosage/threshold dependent.

### Elevated intracellular GTP promotes chloramphenicol's bactericidal effect

**Figure 4:**
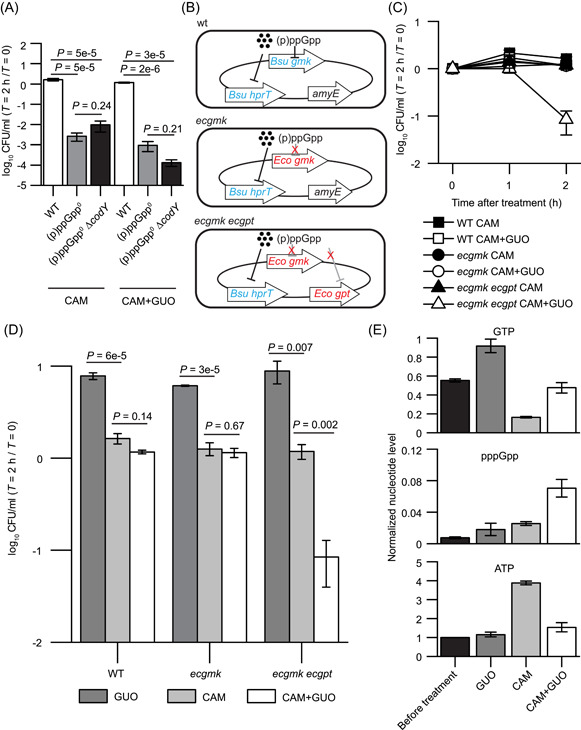
Increasing GTP in wild‐type cells potentiates chloramphenicol (CAM) lethality. (A) Exponential‐phase *Bacillus subtilis* SMY wild‐type, (p)ppGpp^0^, and (p)ppGpp^0^ Δ*codY* cells were treated with 12 μg/ml CAM or 12 μg/ml CAM and 1 mM guanosine (GUO). The relative CFU/ml was determined by dividing the number of CFU/ml at each time point by the number of CFU/ml at *T* = 0. Values are means (*N* ≥ 3) ± SEM. A two‐tailed two‐sample equal‐variance Student's *t* test was performed between samples indicated by *P* values. (B) Schematic of the gene content for *B. subtilis* SMY strains wild type, *ecgmk*, and *ecgmk ecgpt*. Genes in the same color are the same in each strain. (C) Exponential‐phase *B. subtilis* SMY wild type, *ecgmk* and *ecgmk ecgpt* were treated with 12 μg/ml CAM or 12 μg/ml CAM and 1 mM GUO. Relative CFU/ml was determined by dividing the number of CFU/ml at each time point by the number of CFU/ml at *T* = 0 and converted to a percentage. Values are means (*N* ≥ 2) ± SEM. (D) Exponential‐phase *B. subtilis* SMY wild type, *ecgmk*, and *ecgmk ecgpt* were treated with 1 mM GUO, 12 μg/ml CAM or 12 μg/ml CAM, and 1 mM GUO. The relative CFU/ml was determined by dividing the number of CFU/ml at *T* = 2 h by the number of CFU/ml at *T* = 0 and converted into a percentage. Values are means (*N* ≥ 2) ± SEM. A two‐tailed two‐sample equal‐variance Student's *t* test was performed between samples indicated by *P* values. (E) Exponential‐phase *ecgmk ecgpt* was radiolabeled with ^32^P‐orthophosphate and treated with 1mM GUO, 12 μg/ml CAM or 12 μg/ml CAM and 1 mM GUO for 1 h. Nucleotides were extracted and GTP, pppGpp, and ppGpp levels were determined and normalized to the ATP level at *T* = 0 and OD_600_ relative to *T* = 0. Values are means (*N* ≥ 2) ± SEM.

We have shown that lowering GTP is sufficient for chloramphenicol protection even without (p)ppGpp (Figure 3). We next examine whether lowering GTP is also necessary for protection against chloramphenicol. If this hypothesis was true, then high GTP levels, even with (p)ppGpp, should be sufficient for chloramphenicol lethality. We first ruled out the requirement of the GTP‐activated transcription regulator CodY^49^, ^50^ for chloramphenicol killing, as both (p)ppGpp^0^ and (p)ppGpp^0^Δ*codY* were killed by chloramphenicol treatment (Figure 4A), indicating that lethality triggered by chloramphenicol is independent of the CodY regulon. Next, we increased the level of GTP further in (p)ppGpp^0^ and (p)ppGpp^0^ Δ*codY* mutants by the addition of guanosine in the media, which we have previously confirmed to be effective in increasing intracellular GTP levels robustly in cells without (p)ppGpp^32^. The addition of guanosine (GUO) during chloramphenicol treatment further increased its lethality (Figure 4A), confirming that increasing GTP promotes chloramphenicol lethality.

Next, we tested whether abolishing (p)ppGpp's inhibitory effects on GTP biosynthesis has the potential to render chloramphenicol bactericidal even in the presence of sufficiently high levels of cellular (p)ppGpp. This is challenging because (p)ppGpp tightly regulates GTP levels by inhibiting multiple enzymes involved in GTP synthesis, including Gmk, Xpt, and HprT. Therefore, we constructed multiple mutations in *B. subtilis*, leading to (p)ppGpp‐refracting variants of multiple enzymes (Figure 4B). We first constructed a mutant (*ecgmk*) in which the endogenous *B. subtilis gmk* (*gmk*_*Bs*_) (the gene encoding guanylate kinase that phosphorylates GMP to GDP) was replaced with the (p)ppGpp‐insensitive *E. coli gmk* (*ecgmk*)^46^. In addition, we introduced an β‐d‐1‐thiogalactopyranoside (IPTG)‐inducible *E. coli gpt* (*ecgpt*), which encodes xanthine–guanine phosphoribosyltransferase (Gpt), a mildly (p)ppGpp‐resistant *E. coli* variant of HprT (HprT_*Bs*_ half‐maximal inhibitory concentration [IC_50_] = ~10 μM, Gpt_*Ec*_ IC_50_ = 45 μM)^32^, ^43^ to further decrease the inhibitory effects of (p)ppGpp on GTP biosynthesis via the salvage pathway. The resulting *ecgmk ecgpt* strain bypasses (p)ppGpp regulation of Gmk and HprT to allow (p)ppGpp‐refractory GTP biosynthesis via the guanosine salvage pathway (Figure 4B). The addition of guanosine in the growth media can increase GTP levels in *ecgmk ecgpt* mutant cells to much higher concentrations than wild‐type cells or *ecgmk* cells^43^, ^46^.

Using this strain, we determined whether increasing GTP levels sensitize (p)ppGpp+ cells to chloramphenicol. We treated the *ecgmk ecgpt* strain with guanosine to potentiate GTP accumulation concomitant to chloramphenicol treatment (chloramphenicol + guanosine). Strikingly, around 90% of *ecgmk ecgpt* cells were killed by chloramphenicol + guanosine treatment (Figure 4C), although chloramphenicol treatment or guanosine addition alone did not kill the *ecgmk ecgpt* cells, and chloramphenicol + guanosine double treatment did not kill wild‐type cells or the *ecgmk* single mutant (Figure 4D). We quantified cellular nucleotide pools via radiolabeled thin‐layer chromatography (TLC). Treatment of *ecgmk ecgpt* cells with chloramphenicol and guanosine resulted in elevated GTP compared to the *ecgmk* single mutant (Figures S1 and S2), providing complete confirmation of their killing effects. (p)ppGpp also increased after chloramphenicol+ guanosine treatment (Figure 4E). The fact that *ecgmk ecgpt* was extensively killed by chloramphenicol upon GTP elevation despite the presence of increased (p)ppGpp strongly confirms that lowering GTP is the effector downstream of (p)ppGpp and necessary for stasis and prevention of chloramphenicol lethality. Combined with the sufficiency of lowering GTP in surviving chloramphenicol suggested above, these results altogether indicated that (p)ppGpp downregulates GTP levels to prevent chloramphenicol lethality.

### Chloramphenicol pretreatment prevents *Bacillus subtilis* lethality in the presence of a bactericidal antibiotic

**Figure 5:**
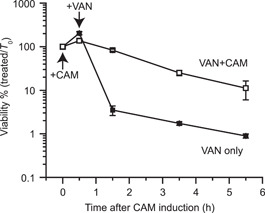
Chloramphenicol (CAM) pretreatment of *B. subtilis* increases survival of bactericidal antibiotic vancomycin (VAN). Log‐phase LB culture of the *B. subtilis* NCIB3610 wild‐type strain was pretreated with 2 μg/ml CAM (+CAM) for 30 min and then treated with 2 μg/ml (20× minimum inhibitory concentration [MIC]) VAN (+VAN). Control culture was not pretreated with CAM (−CAM) and was directly treated with VAN. Percent viability over time was analyzed. Values are means (*N* = 3) ± SEM.

Our results suggest that the bacteriostatic effect of chloramphenicol is not due to it being intrinsically less corruptive to macromolecular processes, but rather due to its ability to induce (p)ppGpp, which protects bacteria against its potential bactericidal effect. If this was the case, then chloramphenicol may condition *B. subtilis* to survive other bactericidal antibiotic treatments. To test this hypothesis, we pretreated wild‐type *B. subtilis* with chloramphenicol, followed by treatment with ~20× MIC of the cell envelope‐targeting bactericidal antibiotic vancomycin and measured its survival over time (Figure 5). In the absence of the chloramphenicol pretreatment, vancomycin killed ~99% of the *B. subtilis* population within the first hour (Figure 5). In contrast, the chloramphenicol‐treated population was highly refractory to vancomycin treatment, with ~90% survival after the first hour of antibiotic addition, and then slowly declined over time (Figure 5). This result suggests that chloramphenicol bacteriostasis is due to its induction of *B. subtilis*'s stringent response, which protects bacteria against the potential bactericidal effects of classical bactericidal antibiotics such as vancomycin, in addition to chloramphenicol itself.

## DISCUSSION

Elucidating how bacteria respond to antibiotics is fundamental to understanding their bactericidal or bacteriostatic effects and clinical applications. Here, we show that induction of (p)ppGpp by the bacteriostatic antibiotic chloramphenicol is important for *B. subtilis* to protect from its lethality. The protective effect of (p)ppGpp from chloramphenicol treatment can be largely attributed to its downstream regulation of GTP synthesis. In addition, the induction of (p)ppGpp by chloramphenicol may enhance survival to subsequent treatment with bactericidal antibiotics. Taken together, our findings suggest that (p)ppGpp is a key determinant for controlling the bacteriostatic and bactericidal activity of ribosome‐targeting antibiotics in Gram‐positive bacteria.

### Chloramphenicol induces (p)ppGpp production in *Bacillus* through RelA

The nucleotide (p)ppGpp is best known as signals that are strongly induced by amino acid starvation, through the activation of ribosome‐associated enzyme RelA by uncharged tRNA. In this study, we showed that *B. subtilis* produces (p)ppGpp in response to chloramphenicol treatment through RelA. This finding corroborates with and provides a mechanistic lead to an unexplained observation almost 50 years ago that translation inhibitors increase (p)ppGpp levels in *B. subtilis* ^33^. Future mechanistic studies are required to decipher how chloramphenicol activates (p)ppGpp synthesis by RelA.

While (p)ppGpp can also be induced by other bacteriostatic drugs such as tetracycline^33^ and mupirocin^51^, not all bacteriostatic antibiotics induce (p)ppGpp. For example, we found that trimethoprim, a bacteriostatic inhibitor of dihydrofolic acid reductase, does not induce (p)ppGpp in *B. subtilis* (Figure S3). Therefore, trimethoprim is likely bacteriostatic via a different mechanism.

In contrast to *B. subtilis*, chloramphenicol does not induce (p)ppGpp and has been reported to reduce starvation‐induced (p)ppGpp in *E. coli* ^52^, ^53^. The species difference in (p)ppGpp production upon the same antibiotic treatment suggests that RelA or its regulation may have evolved to respond to different cues in divergent species that may be linked to their ecological niches. For example, chloramphenicol and tetracycline are natural antibiotics produced by soil microbes such as *Streptomyces* ^54^, which shares the same natural habitat as *Bacillus* ^55^. Chloramphenicol has also been reported to induce *B. subtilis* motility at subinhibitory concentrations^56^. Thus, *Bacillus* may have evolved multiple physiological responses to these antibiotics: stimulated motility to distance from antibiotic producers by sensing their secreted antibiotic at low concentrations to increase available resources^57^, and eliciting a general protective response through (p)ppGpp production when the local antibiotic concentration increases.

### Prevention of chloramphenicol lethality through (p)ppGpp‐regulated GTP homeostasis in *Bacillus*

**Figure 6:**
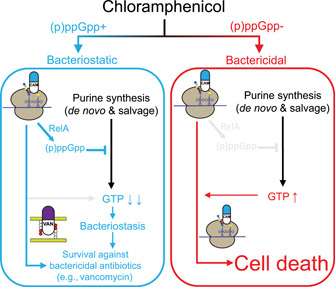
Working model of (p)ppGpp induction by chloramphenicol (CAM) and protection from its bactericidal effect. CAM binds to ribosome to inhibit translation elongation. In wild‐type cells, CAM treatment activates RelA to produce (p)ppGpp. (p)ppGpp inhibits GTP biosynthesis and depletes GTP, promoting bacteriostasis. The bacteriostatic effect of CAM also promotes survival against further treatment with bactericidal antibiotics such as vancomycin. In (p)ppGpp^0^ cells, loss of (p)ppGpp results in GTP dysregulation, which potentiates lethality of the antibiotic, leading to cell death.

Antibiotic lethality is a multifactorial trait that can vary between bacterial species^4^ and growth conditions such as nutrient availability^58^. Chloramphenicol, a classic broad‐spectrum bacteriostatic antibiotic, is bactericidal to some species such as *Haemophilus influenzae* and *Streptococcus pneumoniae* ^59^, ^60^. Here, we found that regulation of GTP biosynthesis enzymes by (p)ppGpp is a key to the prevention of chloramphenicol lethality in *B. subtilis* (Figure 6). Given the conservation of GTP biosynthesis regulations in Firmicutes^32^, ^36^, ^38^, ^43^, ^44^, ^46^, it is possible that similar protection mechanisms also apply to Firmicute pathogens such as *E. faecalis*, in which we also observed (p)ppGpp‐dependent lethality by chloramphenicol (Figure 1D). On the other hand, Gram‐negative bacteria such as *E. coli* do not appear to require (p)ppGpp to survive chloramphenicol^26^, indicating that Gram‐negative bacteria have evolved different protection mechanisms against this antibiotic.

How does GTP dysregulation contribute to chloramphenicol lethality in *Bacillus*? We considered three possibilities: one possibility is that elevated GTP is the cause of chloramphenicol lethality, as we have previously shown that elevating intracellular GTP levels alone can lead to an unexplained “death‐by‐GTP” in (p)ppGpp‐defective *B. subtilis* ^32^. In (p)ppGpp‐defective *E. faecalis*, a Gram‐positive pathogen from the same phylum *as B. subtilis*, increasing GTP levels can stop cells from growing^61^. However, chloramphenicol killing is unlikely mediated entirely through its elevation of GTP. In the case of “death‐by‐GTP”, the level of cellular GTP typically increases by 15‐fold or more^32^, ^43^. In contrast, as low as a two‐fold increase in GTP is sufficient to result in at least 90% killing by chloramphenicol (Figure 3C,D).

The second possibility, which we did not rule out, is that chloramphenicol is in fact lethal. However, the induction of (p)ppGpp lowered GTP, resulting in cell stasis that prevented the bactericidal action of chloramphenicol. This is largely mediated by direct regulation of GTP biosynthesis and transcription factors by (p)ppGpp (Figure 3A). This possibility is supported by the observation that wild‐type cells pretreated with chloramphenicol also survive treatment with bactericidal antibiotics such as vancomycin (Figure 5).

The third hypothesis is that chloramphenicol killing is facilitated by increased levels of GTP. Notably, increasing GTP via guanosine addition has little effect on *ecgmk ecgpt* cells, while addition of chloramphenicol along with guanosine is required for bacterial killing (Figure 4D–E). Thus, an increase in GTP is a key facilitator of the chloramphenicol bactericidal effect, rather than the immediate cause of bacterial death. Further investigation is necessary to understand how an increase in GTP facilitates chloramphenicol lethality, and whether an increase in GTP also promotes lethality of other antibiotics.

### Benefits of targeting (p)ppGpp synthesis to improve antibiotic therapy

We found that chloramphenicol induction of (p)ppGpp not only prevented lethality of chloramphenicol but possibly also conferred protection against subsequent treatment with bactericidal antibiotics such as vancomycin. This phenomenon resembled a classic observation of antibiotic antagonism^62^, where a bacteriostatic agent hinders the killing by another agent in combination therapy. In *Bacillus*, (p)ppGpp induced by chloramphenicol treatment might contribute to the survival of bactericidal antibiotic treatment, resulting in drug antagonism. This phenomenon is not restricted to chloramphenicol, but extends to other bacteriostatic antimicrobial agents such as triclosan^63^. The chloramphenicol‐induced drug antagonism is conserved in *E. coli*, but is not mediated by (p)ppGpp, since *E. coli* does not produce (p)ppGpp in response to chloramphenicol^52^. Nevertheless, in pathogens that produce (p)ppGpp in response to bacteriostatic antibiotics, disrupting (p)ppGpp synthesis as an antimicrobial strategy not only can render bacteriostatic antibiotics such as chloramphenicol bactericidal to enable new treatment options but can also improve combination therapy by reducing antibiotic antagonism.

## MATERIALS AND METHODS

### Strains, growth conditions, and media

The strains used are listed in Table S1. Cell cultures were grown at 37°C with shaking at 250 rpm. Unless stated otherwise, *B. subtilis* strains were grown in a modified S7‐defined medium^64^: MOPS was used at 50 mM rather than 100 mM, supplemented with 0.1% glutamate, 1% glucose, and 0.5% casamino acids. For YB886 background strains, 50 μg/ml tryptophan and 50 μg/ml methionine were supplemented in the medium^65^. For the *ecgmk ecgpt* strain, isopropyl IPTG was added to a final concentration of 0.5 mM to induce *ecgpt* expression from an IPTG‐inducible promoter. *E. faecalis* strains were grown in BHI medium (Sigma‐Aldrich) at 37°C, 250 rpm, under aerobic conditions.

### Strain construction

The plasmids and primers used in strain construction are listed in Tables S2 and S3, respectively. To construct the *relA* ^syn^ mutant, the (p)ppGpp synthetase‐defective allele *relA* ^D264G^ ^32^ was introduced into *B. subtilis* NCIB 3610 pBS32‐(DK847) using the markerless gene replacement protocol. Briefly, plasmid pJW371 (containing an *relA* A791G mutation, which will lead to a D264G substitution in the RelA protein, flanked by two ~400 bp *relA* sequences upstream and downstream, the *amp* and cat selection marker and the I‐*Sce*I endonuclease cut site)^32^ was transformed into DK847 and integrated into the chromosome by a single cross‐over in the *relA* sequence. The resulting strain was subsequently transformed with pSS4332 (which expresses the I‐*Sce*I endonuclease^66^) to induce a double‐strand break at the I‐*Sce*I cut site within the integrated plasmid to induce a recombination event that removes the selection markers. The resulting clones either contain the wild type *relA* or the *relA* ^D264G^ mutation, from which the *relA* ^D264G^ mutants were identified by PCR and DNA sequencing of the *relA* gene using primers oJW418/oJW419. Positive clones were subjected to DNA sequencing verification to obtain the strain JDW2721.

To construct the (p)ppGpp^0^ mutant, the (p)ppGpp synthetase genes *sasB*, *sasA*, and *relA* were sequentially deleted from the *B. subtilis* wild‐type background NCIB 3610 pBS32‐(DK847). The markerless gene replacement process described above was applied to delete *sasB* and *sasA*, using the plasmids pJW300 (containing the region of homology upstream and downstream *sasB* gene, selection markers, and the I‐*Sce*I endonuclease cut site) and pJW306 (containing the region of homology upstream and downstream *sasA* gene, selection markers, and the I‐*Sce*I endonuclease cut site), respectively. The correct recombinants were verified by PCR using primers oJW879/oJW880 (*sasB*) and oJW904/oJW905 (*sasA*), respectively. The sequential markerless deletion yields the Δ*sasB* Δ*sasA* strain JDW2230. Finally, *relA* deletion was performed by transforming a PCR product containing the *relA*::*mls* locus amplified by primers oJW418/oJW419 from TW30^67^ genomic DNA into JDW2230. Successful recombination was verified by PCR. The *relA* ^syn^ and (p)ppGpp^0^ phenotypes were confirmed by plating and checking their inability to grow on S7 + glucose agar without amino acids^32^, to obtain strain JDW2231.

### Isolation of (p)ppGpp^0^ *gmk* ^Q110R^

(p)ppGpp^0^ (JDW2231) was grown on S7 + glucose medium without amino acids. The colonies that grew were all suppressors and most were in the *codY* and *guaB* genes^48^. The colonies were then screened on solid media containing S7 + casamino acids + 0.5 mM 8‐azaguanine^68^ and S7 + casamino acids + 0.1 mM guanosine^32^. Survivors of guanosine, but not 8‐azaguanine, were sequenced to identify the mutant *gmk* allele. (p)ppGpp^0^ *gmk* ^Q110R^ was isolated and confirmed in this suppressor screen.

### Antibiotic killing assay

To evaluate the bactericidal effect of the antibiotics, *B. subtilis* cells were washed from young colonies on LB agar (less than 15 h incubation) and inoculated into S7 liquid medium supplemented with 0.5% casamino acids at an initial OD_600_ = 0.005. Cells in the logarithmic phase (0.2–0.4 OD_600_) were treated with 12 μg/ml chloramphenicol or 0.5 μg/ml tetracycline. Samples were taken at indicated time points before and after treatment, serially diluted with antibiotic‐free medium, and then plated onto LB agar plates. LB agar plates were incubated at 37°C overnight. Colony‐forming units (CFUs) were determined by counting the next‐day colonies and normalized to the liquid sample volume to obtain CFU/ml. Relative CFU/ml was calculated by dividing the CFU/ml at a specific time point by CFU/ml at the time point immediately before drug treatment.

To evaluate the bactericidal effect of chloramphenicol in *E. faecalis*, cultures of wild‐type and (p)ppGpp^0^ *E. faecalis* were grown in BHI medium at 37°C 250 rpm with aeration to the logarithmic phase (OD_600_ = 0.1–0.2). To begin treatment, 25 μg/ml chloramphenicol was added to the culture. Cultures were sampled at different time points after treatment, diluted in BHI medium, plated on BHI agar, and grown at 37°C overnight to determine viability in CFU. Survival was reported as CFU/ml normalized to the CFU/ml immediately before (*T* = 0) treatment.

### MIC measurement

MIC measurements of antibiotics were performed by microdilution^3^ in Mueller–Hinton broth (MHB) (Sigma) for *B. subtilis*, or in BHI medium for *E. faecalis*. Cells were grown in MHB at 37°C 250 rpm to OD_600_ ~0.2, and then diluted to 100 μl in MHB to a final OD_600_ of 0.005 in each well on a 96‐well plate. The wells of the culture contained a gradient of antibiotic concentrations (0, 0.75, 1.5, 3, and 6 μg/ml for chloramphenicol; 0, 1, 2, 4, and 8 μg/ml for tetracycline). The 96‐well plate was incubated at 37°C overnight. The well with the lowest concentration of antibiotics achieving no visible turbidity was recorded, and the corresponding concentration is the MIC.

### Fluorescence microscopy

To differentiate whether antibiotics can induce cell death using microscopy, the Live/Dead BacLight Viability Kit (Invitrogen) was used (3 μl dye mixture per ml cell resuspension, incubating for 15 min), in which live and dead cells are stained with SYTO9 and propidium iodide, respectively. Cells treated with chloramphenicol and stained by the viability kit were trapped in 1% agarose pads infused with an S7‐defined medium. Cells were visualized on an Olympus IX‐83 inverted microscope, with a 60x phase‐contrast objective, using fluorescence filters (excitation: 470/20 nm, dichroic mirror: 485 nm, emission: 515/50 nm for SYTO9; excitation: 575/20 nm, dichroic mirror: 595 nm, emission: 645/90 nm for propidium iodide).

### Measurement of intracellular nucleotides by TLC

Intracellular levels of ATP, GTP and (p)ppGpp were quantified by ^32^P‐radiolabeled TLC. The culture growth, radiolabeling, sample collection, TLC run, and phosphorimaging were performed in the same manner as in reference^45^. Nucleotide levels were quantified by ImageJ (NIH). Nucleotide levels are normalized to relative OD_600_ to time zero and then divided by the ATP level at time zero. For quantification of nucleotide levels, normalized nucleotide levels are multiplied by the ATP concentration at time zero (assume 5 mM based on measurement in previous work^69^) to determine the estimated nucleotide concentrations.

## AUTHOR CONTRIBUTIONS

Jue D. Wang conceptualized the study; Jin Yang, Jessica T. Barra, and Danny K. Fung performed the experiments; Jin Yang, Jessica T. Barra, and Danny K. Fung performed the data analysis; and Jin Yang, Jessica T. Barra, Jue D. Wang, and Danny K. Fung wrote the manuscript.

## ETHICS STATEMENT

No animal or human research was involved.

## CONFLICT OF INTERESTS

The authors declare no conflict of interests.

## Supporting information

Supporting information.

## Data Availability

The data that support the findings of this study are available from the corresponding author upon reasonable request.
