# Supplementary material for: Bacillus subtilis produces (p)ppGpp in response to the bacteriostatic antibiotic chloramphenicol to prevent its potential bactericidal effect
Source: mLife. 2022 Jun 30;1(2):101–13. doi: 10.1002/mlf2.12031 (PMC10989873; doi:10.1002/mlf2.12031)
Supplement: Supplementary file 1 — Supporting information. [file MLF2-1-101-s001.pdf]

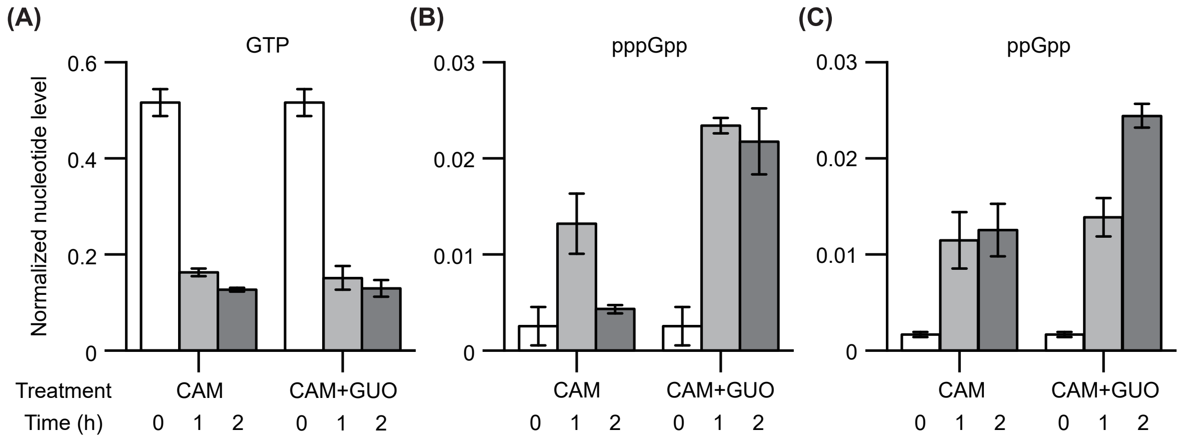

Figure S1. Nucleotide levels in *ecgmk* under chloramphenicol (CAM) and chloramphenicol + guanosine (CAM+GUO) treatment. ^{32}P-labeled orthophosphate was incorporated into early-exponential phase *ecgmk* cells. Cells were treated with 12 µg/ml CAM or 12 µg/ml CAM and 1 mM GUO when mid-exponential phase was reached. At each time point, nucleotides were extracted and analyzed using TLC. GTP (**A**), pppGpp (**B**) and ppGpp (**C**) were calculated, normalized to ATP levels at T = 0 and relative OD_{600} to T = 0. Values are N = 2 ± SEM.

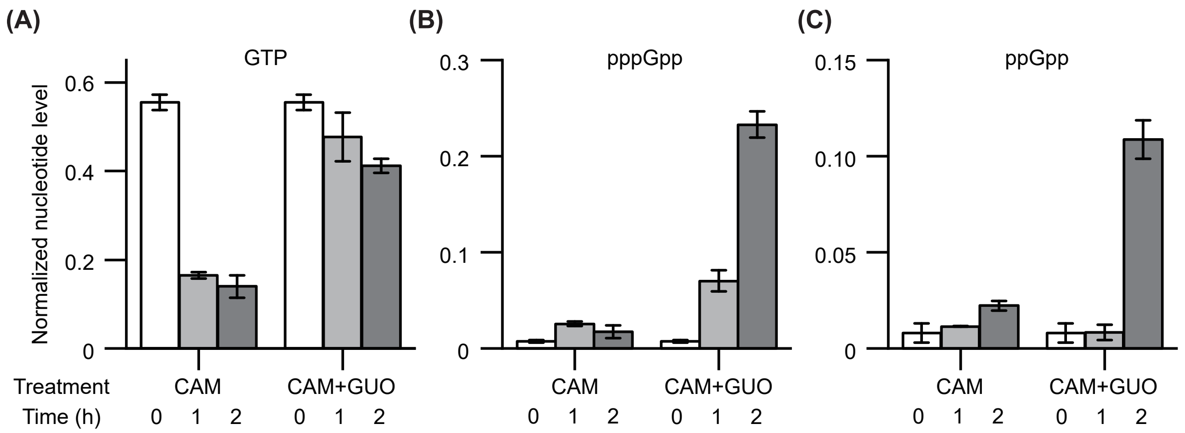

Figure S2. Nucleotide levels in *ecgmk ecgpt* under chloramphenicol (CAM) and chloramphenicol + guanosine (CAM+GUO) treatment. ^{32}P-labeled orthophosphate was incorporated into early-exponential phase *ecgmk ecgpt* cells. Cells were treated with 12 μg/ml CAM or 12 μg/ml CAM and 1 mM GUO when mid-exponential phase was reached. At each time point, nucleotides were extracted and analyzed using TLC. GTP (**A**), pppGpp (**B**) and ppGpp (**C**) were calculated, normalized to ATP levels at *T* = 0 and relative OD_{600} to *T* = 0. Values are *N* = 2 ± SEM.

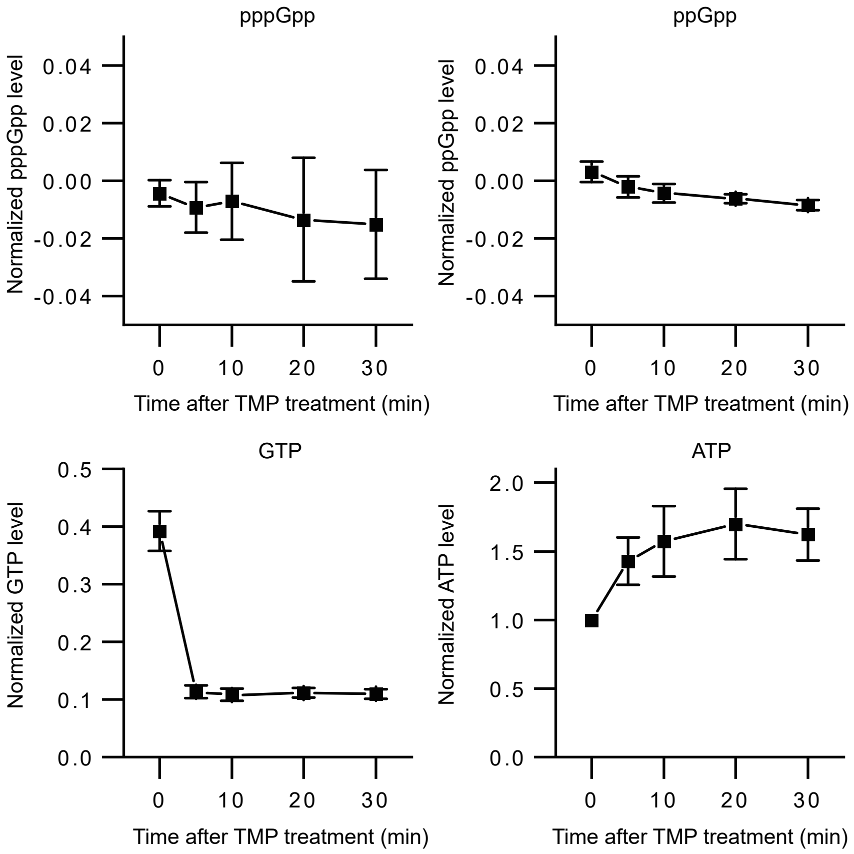

Figure S3. Nucleotide levels in NCIB3610 WT under trimethoprim (TMP) treatment. ^{32}P-labeled orthophosphate was incorporated into early-exponential phase WT cells. Cells were treated with 4x MIC (2 µg/ml) TMP when mid-exponential phase was reached. At each time point, nucleotides were extracted and analyzed using TLC. pppGpp, ppGpp, GTP and ATP were calculated, normalized to ATP levels at T = 0 and relative OD_{600} to T = 0. Values are N = 3 ± SEM.
